# Transcranial magnetic stimulation of visual cortex in migraine patients: a systematic review with meta-analysis

**DOI:** 10.1007/s10194-012-0445-6

**Published:** 2012-04-27

**Authors:** Francesco Brigo, Monica Storti, Raffaele Nardone, Antonio Fiaschi, Luigi Giuseppe Bongiovanni, Frediano Tezzon, Paolo Manganotti

**Affiliations:** 2 Department of Neurological, Neuropsychological, Morphological and Movement Sciences, Section of Clinical Neurology, University of Verona, Piazzale L.A. Scuro 10, 37134 Verona, Italy; 3 Department of Neurology, Franz Tappeiner Hospital, Merano, Italy; 4 Department of Medicine, University of Verona, Verona, Italy; 5 Department of Neurology, Christian Doppler Klinik, Paracelsus Medical University, Salzburg, Austria

**Keywords:** Meta-analysis, Migraine, Phosphenes, Systematic review, Transcranial magnetic stimulation

## Abstract

We systematically reviewed the literature to evaluate the prevalence of phosphenes and the phosphene threshold (PT) values obtained during single-pulse transcranial magnetic stimulation (TMS) in adults with migraine. Controlled studies measuring PT by single-pulse TMS in adults with migraine with or without aura (MA, MwA) were systematically searched. Prevalence of phosphenes and PT values were assessed calculating mean difference (MD) and odds ratio (OR) with 95 % confidence intervals (CI). Ten trials (277 migraine patients and 193 controls) were included. Patients with MA had statistically significant lower PT compared with controls when a circular coil was used (MD −28.33; 95 % CI −36.09 to −20.58); a similar result was found in MwA patients (MD −17.12; 95 % CI −23.81 to −10.43); using a figure-of-eight coil the difference was not statistically significant. There was a significantly higher phosphene prevalence in MA patients compared with control subjects (OR 4.21; 95 % CI 1.18–15.01). No significant differences were found either in phosphene reporting between patients with MwA and controls, or in PT values obtained with a figure-of-eight coil in MA and MwA patients versus controls. Overall considered, these results support the hypothesis of a primary visual cortex hyper-excitability in MA, providing not enough evidence for MwA. A significant statistical heterogeneity reflects clinical and methodological differences across studies, and higher temporal variabilities among PT measurements over time, related to unstable excitability levels. Patients should therefore be evaluated in the true interictal period with an adequate headache-free interval. Furthermore, skull thickness and ovarian cycle should be assessed as possible confounding variables, and sham stimulation should be performed to reduce the rate of false positives. Phosphene prevalence alone cannot be considered a measure of cortical excitability, but should be integrated with PT evaluation.

## Introduction

Several aspects in the pathophysiology of migraine are still unknown, although an altered cortical excitability has been proposed as an important factor predisposing to the spontaneous cortical spreading depression which is thought to represent the pathophysiological basis of the migraine with aura [1]. A generalized cortical inter-ictal hyper-excitability, more pronounced in the visual cortex, has been suggested in migraine [1, 2], although other psychophysical tests of the visual system yielded results suggestive of occipital cortex hypo-excitability or lack of intra-cortical excitation [3]. Neurophysiologic evidence for inter-ictal primary visual cortex hyper-excitability is nevertheless controversial, with some studies demonstrating amplitude differences of visual evoked responses in patients with migraine compared with controls [4–6], and other studies not confirming such results [7, 8]. However, a recent study using VEP with paired pulse stimulation in patients affected by migraine without aura demonstrated a reduced inhibitory response to the second pulse, compatible with a condition of reduced inhibition-increased excitability [9].

Transcranial magnetic stimulation (TMS) has been proposed as an innovative tool to noninvasively and directly assess the cortical physiology and excitability in vivo. In recent years, TMS has been repeatedly used in patients with migraine to test occipital cortex excitability by measuring phosphene threshold (PT), defined as the minimum intensity of a TMS pulse needed to evoke phosphenes: PT is inversely related to the overall level of visual cortex excitability [10], so that a low PT is considered expression of primary visual cortex hyper-excitability. Important discrepancies among different studies do, however, exist, with some groups reporting increased, and others decreased inter-ictal PT. These studies produced conflicting results also regarding prevalence of stimulation-induced phosphenes in migraineurs compared with healthy controls. These discrepancies make it very difficult to reach a definite conclusion by simple summation of previous results. We therefore decided to undertake a systematic review and a meta-analysis of studies evaluating phosphene prevalence and inter-ictal PT values during single-pulse TMS in adults.

Some reviews previously assessed this topic, but always in a narrative and subjective way, not using systematic and explicit methods to identify, select and critically appraise studies, and to extract data, and to analyse them with statistical methods [11–15]. The present review represents therefore the first attempt to appraise the available literature on magneto-phosphenes in migraine with systematic methods.

## Methods

This review was guided by a written pre-specified protocol describing research questions, review methods, plan for data extraction and synthesis.

Our aim was to critically and systematically evaluate the literature to determine (A) the prevalence of phosphenes and (B) the PT values obtained during single-pulse TMS in adults with migraine compared with controls.

We therefore included only controlled studies measuring PT by single-pulse TMS in adults of either gender with migraine (with or without aura; defined according to International Headache Society criteria, 1988 and 2004 [16, 17]) and in control subjects, regardless of stimulator characteristics such as coils’ shape, size and maximum magnetic field strength. Uncontrolled studies, studies conducted in children or performing TMS with paired magnetic stimuli, or stimulating cortical regions other than primary visual cortex were excluded. Children were excluded in order to exclude an excessively high clinical heterogeneity (related to age differences) and methodological heterogeneity (e.g. related to the use of smaller TMS coils, or with lower stimulation intensity in this population). Studies performing TMS with paired magnetic stimuli were excluded to prevent a high heterogeneity due to the use of two different methods. Also consecutive TMS studies were excluded.

The MEDLINE (accessed by Pubmed; 1966–June 2011) and EMBASE (1988–June 2011) electronic databases were searched using the following medical subject headings (MeSH): “Phosphenes”, “Transcranial Magnetic Stimulation” and “Migraine Disorders”, as well as following free terms, combined in multiple search strategies with Boolean operators (see “Appendix”) in order to find relevant articles: “migrain*”, “phosphen*”, “phosphene threshold”. Furthermore, all references lists in identified trials were scrutinized for studies not indexed in the electronic databases. In order to provide a transparency of results as great as possible, and to allow readers to reproduce the methodology we adopted, and considering that in abstracts many methodological aspects are not declared and results are often synthesized, only in extenso papers and articles already published were considered eligible for inclusion.

Following data were extracted: inclusion/exclusion criteria, number and sex of participants, headache-free interval, menstrual phase, stimulator characteristics, blinding, definition of PT, and inter-stimulus interval. Data were independently extracted by two review authors (FB, MS) and cross-checked. All disagreements were resolved by consensus. Although we did not systematically evaluate the inter-rater agreement for the data extraction, consensus reached through discussion ensured unanimous decisions.

In case of missing or incomplete data, principal investigators of included trials were contacted and additional information requested.

Two review authors (FB, MS) independently assessed the methodological quality of each study and risk of bias, focusing on blinding and other potential sources of bias.

Provided we thought it clinically appropriate, and no important clinical and methodological heterogeneity was found, we summarized results in a meta-analysis.

In order to minimize methodological heterogeneity between studies evaluating PT values, we separately analyzed results from studies using a circular and a figure-of-eight coil; moreover, to reduce clinical heterogeneity, in each outcome (phosphene prevalence; PT values), we separately analyzed data on migraine with aura from data migraine without aura.

Phosphene reporting after TMS procedure (dichotomous data) was analyzed by calculating odds ratio (OR) for each study, with the uncertainty in each trial being expressed using 95 % confidence intervals (CI).

PT values (continuous data) were analyzed by calculating the mean difference for each trial, with the uncertainty in each study being expressed using 95 % CI. For PT values, total of events in each group was the number of participants reporting phosphenes. A weighted effect across studies was also calculated. In the evaluation of PT, we planned also to perform an individual patient data meta-analysis including subjects not reporting phosphenes as bearing a 100 % threshold.

Homogeneity among study results was evaluated using a standard Chi squared test, combined with the *I* ^2^ statistics, and the hypothesis of homogeneity was rejected if the *p* value was less than 0.10. The interpretation of *I* ^2^ for heterogeneity was made as follows: 0–25 % represents low heterogeneity, 25–50 % moderate heterogeneity, 50–75 % substantial heterogeneity, 75–100 % high heterogeneity [18]. Phosphene prevalence and PT values were combined to obtain a summary estimate of value (and the corresponding CI) using a random-effect model. Random-effects model is considered more conservative than a fixed-effect model, since it takes into account the variability between studies, thus leading to wider CIs.

Statistical analyses were undertaken with the Review Manager software developed by the Cochrane Collaboration (5.1).

## Results

### Description of included studies (Table 1)

The search strategy described above yielded 113 results (78 MEDLINE, 31 EMBASE, 3 in reference lists, 1 unpublished study).

**Table 1:** Characteristics of included studies

Study	Inclusion criteria	Exclusion criteria	Diagnosis (no. of subjects, female/male)	Diagnosis, age (years, mean ± SD)	Headache-free interval		Menstrual phase	Equipment, stimulator, Co/MF/EF/CD	Blinding	Definition of PT	Interstimulus interval
					Before TMS	After TMS					
Áfra et al. [37]	M: diagnosis according to IHS (14). C: healthy subjects	M: drugs altering CNS excitability. C: not reported	MA (18, –) MwA (22, –) C (19, –)	MA – MwA – C –	≥3 days	≥3 days	–	Magstim 200 Ci/2.5/–/130	–	Intensity gradually increase until visual experience was reported	–
Aurora et al. [29]	M: diagnosis according to IHS (14). C: healthy subjects	M: drugs altering CNS excitability. C: not reported	MA (11, 10/1) C (11, 8/3)	MA 37 ± 7 C 36 ± 7	≥1 week	–	–	Cadwell MES 10 Ci/2.0/530/95	Study participants	Intensity gradually increase until visual experience was reported	20 s
Aurora et al. [30]	M: diagnosis according to IHS (14). C: healthy subjects	M: drugs altering CNS excitability. C: not reported	MA (14, –) MwA (1, –) C (8, 5/3)	M 39.9 ± 8.2 C 37.3 ± 6.1	≥1 week	–	–	Cadwell MES 10 Ci/2.0/530/95	Study participants	Intensity gradually increase until visual experience was reported	20 s
Mulleners et al. [36]	M: diagnosis according to IHS (14); ≥2 attacks/month in the 3 months before the study. C: healthy subjects	M: contraindication for TMS, any neurologic or ophthalmologic condition other than refractive error; drugs altering CNS excitability. C: lifetime history of >2 attacks of migraine and migraines in the past year	MA (16, 14/2) MwA (12, 6/6) C (16, 14/2)	MA – MwA – C –	≥24 h	–	–	Magstim 200 Ci/2.0/530/130	Investigators not blinded	Intensity gradually increase until visual experience was reported	≥5 s
Bohotin et al. [32]	M: diagnosis according to IHS (14). C: healthy subjects	M: no other medical condition; personal or family history of epilepsy; prophylactic anti-migraine treatment within the 3 months before the study. C: no other medical condition; personal or family history of epilepsy	MA (10, –) MwA (20, –) C (24, 14/10)	M 33.5 ± 10.8 C 23.5 ± 2.5	≥3 days	≥3 days	TMS performed 12–16 days after the firsdt day of menses (at mid-cycle)	Magstim Rapid E/1.2/–/70	–	Lowest intensity (%) able to evoke PP in at least 3 out of 5 trials	–
Aurora et al. [31]	M: diagnosis according to IHS (14). C: healthy subjects	M: >1 muscular contraction headache/month, history of seizures, pacemakers; drugs altering CNS excitability. C: not reported	MA (10, 9/1) MwA (10, 8/2) C (10, 8/2)	MA 38 ± 13 MwA 39 ± 10 C 37 ± 9	≥1 week	–	–	Cadwell Magstim Ci/2.0/530/95	Investigator performing TMS and study participants	Intensity gradually increase until visual experience was reported	20 s
Bohotin et al. [33]	M: diagnosis according to IHS (14). C: healthy subjects	M: neurological, ophthalmological or systemic disorder; personal or family history of epilepsy; prophylactic anti-migraine treatment within the 3 months before the study. C: neurological, ophthalmological or systemic disorder; personal or family history of epilepsy; personal or family history of migraine	MA (13, –) MwA (24, –) C (33, 18/15)	M 30.3 ± 10.1 C 25.5 ± 6.6	≥3 days	≥3 days	TMS performed 12–16 days after the firsdt day of menses (at mid-cycle)	Magstim Rapid E/1.2/–/70	Investigator performing TMS	Lowest intensity (%) able to evoke PP in at least 3 out of 5 trials	–
Gerwig et al. [35]	M: diagnosis according to IHS (15) C: healthy subjects	M: acute neurological illness such as epilepsy, organic mental disorder, or alcohol and substance abuse; drugs altering CNS excitability. C: drugs altering CNS excitability; family history of migraine	MA (19, 12/7) MwA (19, 15/4) C (22, 11/11)	MA 32 ± 8 MwA 39 ± 10 C 30 ± 4	≥3 days	≥3 days	TMS performed during both menstrual phases	Medtronic Dantec MagPro E/–/–/100	Investigator performing TMS	Intensity (%) able to evoke PP in at least 5 out of 10 trials	≥10 s
Gunaydin et al. [34]	M: diagnosis according to IHS (14). C: healthy subjects.	M: drugs altering CNS excitability. C: not reported.	MA (15, 14/1) MwA (15,12/3) C 30 (26/4)	MA 33.9 ± 5.9 MwA 33.0 ± 4.3 C 33.0 ± 4.9	≥1 week	3 days	–	Magstim 200 Ci/–/–/135	Investigator performing TMS and study participants	Intensity gradually increase until visual experience was reported	–
Khedr et al. [38]	M: diagnosis according to IHS (14). C: healthy subjects.	M: <1 attack of migraine/week; patients taking any drug within 24 h before the study. C: family history of migraine; subjects taking any drug within 24 h before the study	MA (18, –) MwA (10, –) C (20, –)	M 33.7 ± 6.9 C 30.5 ± 7.8	≥3 days	≥3 days	Females not tested pre or during menstrual phase	Maglite r 25 E/–/–/90	–	Intensity (%) able to evoke PP in 5 out of 10 trials	≥5 s

*C* controls, *CD* outer coil diameter (mm), *Ci* circular coil, *Co* coil shape, *E* figure-of-eight coil, *EF* electric field strength (V/m), *IHS* International Headache Society, *M* migraine patients, *MA* migraine with aura, *MF* magnetic field strength (Tesla), *MwA* migraine without aura, *PP* phosphenes

Twenty studies were provisionally selected. We excluded ten studies after reading the full published articles: one study was excluded because paired magnetic stimuli were used to induce phosphenes [19]; in one study TMS was applied laterally over visual area V5, and not over primary visual cortex [20]; two studies used a consecutive TMS procedure [21, 22]. One study, conducted on patients with episodic migraine and on patients with ‘probable chronic migraine’, was provisionally included in this review [23], but later excluded because of the lack of further information on migraine features (e.g. presence of aura), and in order to avoid an excessively high clinical heterogeneity among studies. One unpublished study [24] and four studies published as abstracts [25–28].

Thus 10 trials, comprising 277 migraine patients and 193 control subjects, contributed to this review [29–38]: the earliest was published in 1998 and the most recent in 2006. Five studies were conducted by two different groups with common authors (three by Aurora [29–31], two by Bohoyin [32, 33]). One study performing repetitive TMS was included in the review because PT was identified using single-pulse TMS [32].

More detailed characteristics of included studies are reported in Table 1.

### Risk of bias in included studies

Five studies were conducted by groups with common authors and published within a few years apart [29–33], so that the probability of overlapping cases and/or controls could not be ruled out, also because, although contacted, authors did not clarify such an aspect. Thus, it was impossible to determine whether some included papers represent duplicate publications of one study or two separate studies (multiple publication bias). The inclusion of duplicated data may therefore have lead to overestimation of results from these studies.

Two studies reported that the investigator was blinded to the diagnostic subtype of migraine [33, 34]. In two studies, the investigators were reported as blinded to the diagnoses [31, 35], so that it was possible that they knew which participants were controls and which were migraineurs. In one study, the investigators were not blinded regarding headache status [36]. In four studies, the subjects were not informed of what to expect, but were asked to report all sensations they experienced [29–31, 34]; in six studies, the participants were asked to report any bright stimuli appearing in their visual field [32, 33, 35–38].

Four out of 10 studies defined PT reporting the percentage or number of trials where subjects report phosphenes [32, 33, 35, 38].

## Quantitative synthesis

### Phosphene prevalence (Fig. 1)

Results of a study reporting only percentages of phosphene prevalence [37] were not included in the meta-analysis. Despite our intentions, it was impossible to perform an additional individual patient meta-analysis including participants not reporting phosphenes as bearing a 100 % threshold, because included studies reported only mean and SD of subjects reporting phosphenes, not reporting rough data for each participant.

**Fig. 1:**
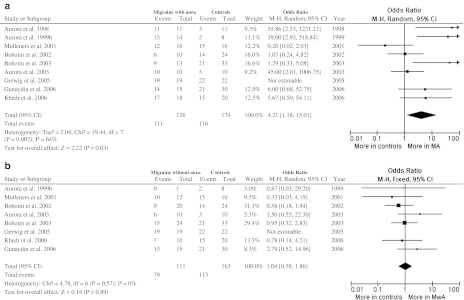
Phosphene prevalence. **a** Participants with migraine with aura (MA); **b** participants with migraine without aura (MwA)

#### Migraine with aura (Fig. 1a)

There were 9 studies with 300 participants. Significant statistical heterogeneity among trials was detected. There was a statistically significant difference in phosphene reporting between migraine with aura and control group, with higher prevalence in migraine group (111/126 vs. 116/174 participants; OR 4.21; 95 % CI 1.18–15.01).

#### Migraine without aura (Fig. 1b)

There were 8 studies with 274 participants. No significant statistical heterogeneity among trials was detected. There was no statistically significant difference in phosphene reporting between migraine without aura and control group (79/111 vs. 113/163 participants; OR 1.04; 95 % CI 0.58–1.86).

### PT values (Fig. 2)

#### Migraine with aura

##### Figure-of-eight coil (Fig. 2a)

There were 4 studies with 123 participants. Significant statistical heterogeneity among trials was detected. There was no statistically significant difference in phosphene threshold between migraine with aura and control group (mean difference: 2.05; 95 % CI −12.18 to 16.29).

**Fig. 2:**
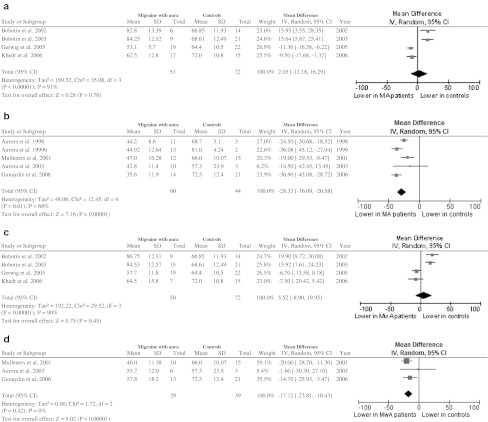
PT values. **a** Patients with MA versus controls (figure-of-eight coil); **b** patients with MwA versus controls (figure-of-eight coil); **c** patients with MA versus controls (circular coil); **d** patients with MwA versus controls (circular coil). Total of events in each group was the number of participants reporting phosphenes. Standard deviations in Mulleners et al. [36] were calculated from standard error and number of participants in each group (standard error × √number of participants)

##### Circular coil (Fig. 2b)

There were 5 studies with 104 participants. Significant statistical heterogeneity among trials was detected. There was a statistically significant difference between groups, with PT being lower in patients with migraine with aura than in controls (mean difference: −28.33; 95 % CI −36.09 to −20.58).

#### Migraine without aura

##### Figure-of-eight coil (Fig. 2c)

There were 4 studies with 122 participants. Significant statistical heterogeneity among trials was detected. There was no statistically significant difference in phosphene threshold between migraine without aura and control group (mean difference: 5.52; 95 % CI −8.90 to 19.95).

##### Circular coil (Fig. 2d)

There were 3 studies with 68 participants. No significant statistical heterogeneity among trials was found. There was a statistically significant difference in phosphene threshold between migraine without aura and control group (mean difference −17.12; 95 % CI −23.81 to −10.43).

## Discussion

In this systematic review, we used systematic and explicit methods to identify, select and critically appraise studies, and to extract data, analyzing them with a meta-analysis. A meta-analysis is the statistical combination of results from two or more separate studies (pair-wise comparisons of interventions), allowing an increase in statistical power, an improvement in precision, sometimes permitting to answer questions not posed by individual studies and to settle controversies arising from conflicting claims.

In the present meta-analysis, we found that patients with migraine with and without aura have a lower PT compared with controls when a circular coil is used; with a figure-of-eight coil the difference is not statistically significant. There was also a statistically significant higher phosphene prevalence in migraine with aura compared with controls. No statistically significant difference was found either in phosphene reporting between patients with migraine without aura and controls, or in PT values obtained by figure-of-eight coil TMS in subjects with migraine with/without aura versus controls. Overall considered (and also taking into account the sample size of each comparison), these results support the hypothesis of a primary visual cortex hyper-excitability in migraine with aura, providing not enough evidence for occipital hyper-excitability in migraine without aura.

In the evaluation of PT, we also planned to perform an individual patient data meta-analysis including subjects not reporting phosphenes as bearing a 100 % threshold. As a matter of fact, if phosphene prevalence is used as a measure of cortical excitability, then subjects not reporting phosphene should be considered as patients with a 100 % threshold. However, included studies reported mean and SD data obtained only in subjects reporting phosphenes, without reporting rough data for each participant, thus preventing us from performing an analysis based on individual subjects data.

How to consider subjects not experiencing phosphenes at 100 % stimulator intensity is still a matter of debate, since the unresponsiveness to magnetic stimulation may be attributed also to specific anatomical peculiarities.

Indeed in most published studies of PTs, there are a certain number of participants who do not experience phosphenes even at maximum stimulator output. There are some anatomical and methodological reasons which may contribute to explain such a phenomenon [39].

First, it is possible that due to methodological difficulties in mapping PT over each square millimeter of the occipital skull, the correct point of stimulation may not be identified in each subject. Second, unlike primary motor cortex, primary visual cortex (calcarine fissure) is deeply located, lying in mid-sagittal plane, so that magnetic field strength applied over the skull may be insufficient to reach and stimulate the visual cortex. Finally, every millimeter the surface cortex is away from the stimulating coil, approximately an additional 3 % of the maximum power output is required to induce an equivalent level of brain stimulation at the motor cortex (although no similar data on visual cortex stimulation is available in the literature).

Hence, although the unresponsiveness to magnetic stimulation might indicate an extremely low visual cortical excitability (so that subjects not reporting phosphene should be considered as patients with a 100 % threshold), it should be taken into consideration that TMS cannot effectively reach deeply located cortical areas. Phosphene prevalence should therefore be interpreted cautiously: as the unresponsiveness to magnetic stimulation depends not only on cortical excitability levels, but also on anatomical features, phosphene prevalence alone cannot be considered as a measure of cortical excitability, but its evaluation should be integrated with PT values.

## Exploration of heterogeneity

The results of the present meta-analysis should be read with cautiousness, mainly because of the considerable statistical heterogeneity found in four out of six meta-analytic comparisons, indicative of inconsistency in the results of included studies. The term “statistical heterogeneity” describes the degree of variation in the effect estimates from a set of studies, and indicates the presence of variability among studies beyond the amount expected due solely to the play of chance.

Such a statistical heterogeneity may be explained both by differences in clinical characteristics of study participants (clinical heterogeneity) and by different stimulation procedures (methodological heterogeneity) adopted.

### Clinical heterogeneity

Regarding clinical heterogeneity, a first aspect to be considered is that, unlike motor TMS, occipital TMS with measuring of PT is a highly subjective procedure, in which different individual attitudes toward detecting, recognizing and reporting phosphenes may play a relevant role. Moreover, the definition of phosphene may be difficult for subjects to understand. Using too detailed definitions or providing too exhaustive instruction may easily introduce biases, and conversely there is the risk that other visual phenomena unrelated to the magnetic stimulus are misinterpreted as phosphenes (risk of false positives). Owing to the subjective nature of phosphenes, a sham stimulation should be added to the standard occipital TMS to reduce the risk of false positives. Sham stimulation may be performed by holding the coil perpendicular to the skull surface resting on the edge; this procedure does not induce a current of sufficient intensity to elicit phosphenes, however, it still provides a comparable acoustic stimulation and sensory percept [40]. This procedure may compensate for the fact that the measurement of phosphenes is subjective by nature, in contrast to the objective measurement of MEPs.

Neuronal excitability of the visual cortex in migraine is not stable but changes relative to the time of the last/next migraine attack [41–44]. Patients should therefore be evaluated in the true interictal period with an adequate headache-free interval (at least 24 h) both before and after the TMS procedure in order to study a homogeneous sample. The variations of cortical excitability might represent the main reason for the data heterogeneity found in the available literature on such a topic. Six out of ten studies in the present review checked and ruled out the occurrence of a migraine attack at least 24 h after TMS recording [32–35, 37, 38]. However, it is worth reporting that even in comparisons between studies performed with an adequate headache-free interval and adopting the same TMS procedures, a significant statistical heterogeneity exists (Fig. 2a, c). This may suggest either that variations of cortical excitability alone do not explain the heterogeneity of studies’ results, or that even among patient studies within the same headache-free interval other factors responsible for clinical heterogeneity exist.

Regarding this last aspect, inter-individual variability in the anatomy of the occipital region and in skull thickness may represent another potentially relevant source of clinical heterogeneity and, to some extent, may be responsible for the impossibility of perceiving phosphenes by some subjects, even when maximum output stimulation is performed [39]. Furthermore, the ovarian cycle may represent another factor influencing cortical excitability [45, 46]; only four studies explicitly assessed such a variable [32, 33, 35, 38], whose effect on cortical excitability is nevertheless still matter of debate [47, 48].

### Methodological heterogeneity

Regarding methodological heterogeneity, potentially relevant aspects to be taken into account are the coils’ shape, size and maximum magnetic field strength, and the direction of the current through the stimulating coil (mono- or bi-phasic); these stimulator characteristics are sometimes not explicitly reported in the studies, although they may deeply influence the results. For example, compared with a figure-of-eight coil, a circular coil stimulates a larger cortical area [49, 50], and may generate, at least theoretically, a stronger electric current resulting in a greater probability of evoking phosphenes.

Another potentially relevant source of methodological heterogeneity is due to discrepancies in PT definitions, and, more in general, to the lack of a unique, systematic TMS protocol in the evaluation of PT in migraine.

Especially in comparison, B (phosphene threshold values evaluated by means of figure-of-eight coil) heterogeneity seems to be related to the presence of some outlying studies with results that conflict with the rest of the studies. Repeating pooled analyses on PT values obtained with figure-of-eight coil and excluding one study at a time to ensure that the results were not skewed by a single (or a few) outlier, it may be easily demonstrated that both the studies conducted by Bohotin [32, 33] are responsible for the greatest amount of statistical inconsistency among studies (mean difference −10.80; CI −15.11 to −6.48 with *I* ^2^ = 0 %). The limited number of included studies prevented us from performing a more detailed sensitivity analysis. We did not find any apparent obvious reason (in terms of clinical and/or methodological differences) for the outlying results.

Such a high degree of inconsistency among meta-analysis may be therefore explained not only by clinical or methodological differences among studies, but also by significant variations of cortical excitability among migraine patients. A higher temporal variability among PT measurements over time [21, 22], related to unstable excitability levels in these patients [22, 41–44], might represent the most relevant clinical factor explaining the high inconsistency among study results found in the present review.

## Conclusions

In this review, we found that patients with migraine with and without aura have a lower PT compared with controls when a circular coil single-pulse TMS is used; with a figure-of-eight coil the difference is not statistically significant. There was also a statistically significant higher phosphene prevalence in migraine with aura compared with controls. No statistically significant difference was found either in phosphene reporting between patients with migraine without aura and controls, or in PT values obtained by figure-of-eight coil TMS in subjects with migraine with/without aura versus controls.

Overall considered, these results support the hypothesis of a primary visual cortex hyper-excitability in migraine with aura, providing not enough evidence for occipital hyper-excitability in migraine without aura.

A significant statistical heterogeneity reflects the presence of clinical and methodological differences across studies, and higher temporal variabilities among PT measurements over time, related to unstable excitability levels in these patients. A unique, shared protocol for future studies of the PT in migraine patients might overcome such limitations. Patients should be evaluated in the true interictal period with an adequate headache-free interval (at least 24 h) both before and after the TMS procedure in order to study a homogeneous sample. Furthermore, skull thickness and ovarian cycle should be assessed as possible confounding variables, and sham stimulation should be performed to reduce the rate of false positives. Since the unresponsiveness to magnetic stimulation depends on cortical excitability levels and on anatomical peculiarities, phosphene prevalence alone cannot be considered as an appropriate measure of cortical excitability, but should be integrated with the parameter of PT. Further studies conducted with a systematic TMS protocol are required to confirm whether and to what extent PT values are reduced in migraine.

## Appendix: search strategies used in the review

### MEDLINE

((“Migraine Disorders”[Mesh]) OR migrain* OR migraine) AND (“Phosphenes”[Mesh]) OR phosphen* OR “phosphene threshold”) AND “Transcranial Magnetic Stimulation”[Mesh])): 78 results.

### EMBASE

(migrain* AND phosphen* AND “Transcranial Magnetic Stimulation”): 31 results.
